# Olfactomedin-1 Has a V-shaped Disulfide-linked Tetrameric Structure*[Fn FN2]

**DOI:** 10.1074/jbc.M115.653485

**Published:** 2015-04-21

**Authors:** Matti F. Pronker, Trusanne G. A. A. Bos, Thomas H. Sharp, Dominique M. E. Thies-Weesie, Bert J. C. Janssen

**Affiliations:** From the ‡ Crystal and Structural Chemistry, Bijvoet Center for Biomolecular Research and; ¶ Van't Hoff Laboratory for Physical and Colloid Chemistry, Debye Institute of Nanomaterials Science, Department of Chemistry, Faculty of Science, Utrecht University, 3584 CH Utrecht, The Netherlands and; § Section Electron Microscopy, Department of Molecular Cell Biology, Leiden University Medical Center, 2300 RC Leiden, The Netherlands

**Keywords:** cell signaling, development, disulfide, electron tomography, neurobiology, small angle x-ray scattering (SAXS), x-ray crystallography, olfactomedin-1 (Olfm1), analytical ultracentrifugation, coiled coil

## Abstract

Olfactomedin-1 (Olfm1; also known as noelin and pancortin) is a member of the olfactomedin domain-containing superfamily and a highly expressed neuronal glycoprotein important for nervous system development. It binds a number of secreted proteins and cell surface-bound receptors to induce cell signaling processes. Using a combined approach of x-ray crystallography, solution scattering, analytical ultracentrifugation, and electron microscopy we determined that full-length Olfm1 forms disulfide-linked tetramers with a distinctive V-shaped architecture. The base of the “V” is formed by two disulfide-linked dimeric N-terminal domains. Each of the two V legs consists of a parallel dimeric disulfide-linked coiled coil with a C-terminal β-propeller dimer at the tips. This agrees with our crystal structure of a C-terminal coiled-coil segment and β-propeller combination (Olfm1^coil-Olf^) that reveals a disulfide-linked dimeric arrangement with the β-propeller top faces in an outward exposed orientation. Similar to its family member myocilin, Olfm1 is stabilized by calcium. The dimer-of-dimers architecture suggests a role for Olfm1 in clustering receptors to regulate signaling and sheds light on the conformation of several other olfactomedin domain family members.

## Teaser

**Background:** Olfactomedin-1 (Olfm1) is a secreted protein with diverse roles in the developing nervous system.

**Results:** We provide the crystal structure of the disulfide-linked coiled coil and olfactomedin domain of Olfm1 and its full-length quaternary arrangement.

**Conclusion:** Olfm1 forms disulfide-linked homotetramers with a V-shaped architecture and binds calcium.

**Significance:** This arrangement suggests a role in receptor clustering and ion channel regulation.

## Introduction

Olfm1^2^ is a secreted oligomerized glycoprotein highly expressed in the developing and adult nervous system (1). It is involved in signaling processes that regulate neuronal development. It has been shown to stimulate neurogenesis, and it influences the timing of neuronal differentiation in several vertebrates (2, 3). Olfm1 also modulates cortical cell migration and neural crest formation (4, 5), and Olfm1 has been recognized as a regulator of axon growth and elongation (6, 7). More recently, aberrant Olfm1 function was also linked to mouse behavioral abnormalities (8). These results suggest that Olfm1 is an important signaling protein in the developing and adult nervous system.

It is not known how Olfm1 regulates these processes, but it has been shown that Olfm1 interacts with a number of proteins that are implicated in these neuronal signaling processes. Olfm1 binds to secreted Wnt inhibitory factor 1, receptors Nogo receptor 1 and amyloid precursor protein, and neuronal cation channel proteins GluR2 AMPA receptor and Cav2.1 voltage-gated calcium channel. These proteins are part of several signaling pathways, and interfering with these interactions by mutation, overexpression, or knockdown of Olfm1 leads to neuronal developmental defects (4, 6, 7). Mutation of Olfm1 disrupts interactions with the AMPA receptor and Cav2.1 channel and increases intracellular calcium levels, resulting in a multitude of signaling malfunctions that culminates in brain dystrophy and behavioral changes (8). How Olfm1 interacts with this diverse set of proteins and how this leads to signaling events that control neuronal developments are not clear.

Olfm1 is a member of the olfactomedin superfamily of which the conserved olfactomedin domain is a defining feature (9, 10). Other well known members are the Olfm1 paralogs Olfm2, Olfm3, and Olfm4 (56, 67, and 22% sequence identity, respectively); gliomedin; myocilin; and latrophilin1, -2, and -3. These proteins have been shown to bind to ligands on the cell surface. The olfactomedin domain is often implicated in interaction with ligands, and many members are believed to be oligomerized. The four Olfm paralogs and myocilin are oligomerized through coiled-coil domains, whereas gliomedin is oligomerized through a collagen-like domain. Oligomerization of these molecules is important for function (10), and Olfm1 can form complexes with Olfm2 and Olfm3 (11, 12). However, it is not clear how Olfm1 or the paralog (Olfm2, -3, and -4) oligomers are structurally arranged.

**FIGURE 1.**
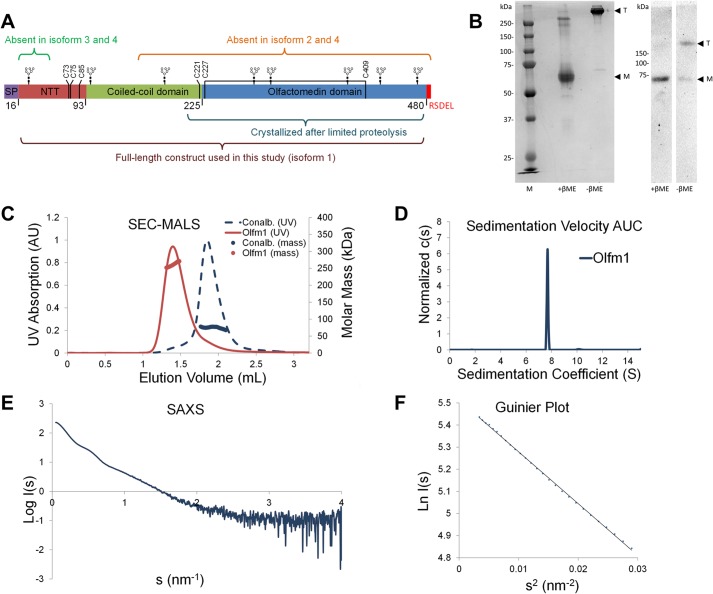
**Olfm1 forms disulfide-linked tetramers.** *A*, isoforms and domains of Olfm1. In this study isoform 1 was used. Cysteines are shown as *vertical black lines. SP*, signal peptide. *B*, non-reducing SDS-PAGE (*left*, Coomassie-stained purified protein; *right*, Western blotted expression medium) shows a shift to >250 kDa, whereas reducing SDS-PAGE shows Olfm1 running at the expected mass of 64 kDa for a (fully glycosylated) monomer (*M*). *T*, tetramer; β*ME*, β-mercaptoethanol; *lane M*, molecular mass markers. *C*, SEC-MALS shows a mass of ∼260 kDa for the Olfm1 peak, which elutes before conalbumin (*Conalb.*) (78 kDa). The plotted UV signal was recorded at 280 nm. *AU*, absorbance units. *D*, AUC sedimentation velocity experiments give a mass of 242 kDa, a sedimentation coefficient of 7.67 S, and a frictional ratio of 1.98 (root mean square deviation, 0.005). *E*, SAXS log *I*(*s*) *versus s* plot of Olfm1 at 3.55 mg/ml. *F*, Guinier plot from SAXS curve agrees with a tetramer (*I*_0_ = 249, *R_g_* = 8.5 nm).

As a result of alternative splicing, four isoforms of Olfm1 are differentially expressed during development (see Fig. 1*A*) (13). All four isoforms of Olfm1 are known to be present in the endoplasmic reticulum as well as being secreted despite isoforms 1 and 3 having a C-terminal -RSDEL quasi-endoplasmic reticulum retention motif (3, 4). The most complete isoform of Olfm1, isoform 1 (also called BMZ), is most similar to the other Olfm paralogs. This isoform comprises a disulfide-containing C-terminal olfactomedin domain, which has recently been shown to be a five-bladed β-propeller in myocilin, gliomedin, and latrophilin3 (14–16). The olfactomedin domain is preceded by a conserved cysteine and a 132-residue-long coiled-coil domain. N-terminal of the coiled-coil domain is a 77-residue large region that contains three conserved cysteines in a C*X*C*X*_9_C arrangement. The first two cysteines (Cys^73^ and Cys^75^) have been found to be essential for Olfm1 oligomerization (17). In isoforms 2 and 4, the C-terminal β-propeller domain and a large part of the coiled coil are absent, whereas isoforms 3 and 4 have an alternative signal sequence and lack the first 35 amino acids of mature Olfm1 (see Fig. 1*A*). Thus, essentially isoform 1 represents the full mature Olfm1 protein, whereas the other three isoforms are lacking regions at the termini.

The structure of Olfm1 and its paralogs is not known. It is not clear how the domains are arranged, which interactions mediate oligomerization, or whether it adopts a defined quaternary structure. Olfm1 interacts with a diverse set of proteins for its signaling functions, but how it performs these various roles is unknown. Lack of structural data has hampered progress in the field. In this study, we determined the structure of the olfactomedin domain of Olfm1 and the quaternary arrangement and architecture of the full-length protein using a combined approach of x-ray crystallography, electron microscopy, and biophysical characterization.

## Experimental Procedures

### 

#### 

##### Protein Expression and Purification

Mouse Olfm1 (NCBI Reference Sequence NP_062371) residues 17–478 (isoform 1) from cDNA IRAVp968C0174D (Source Bioscience) were subcloned using BamHI/NotI sites in pUPE107.03 (N-terminal cystatin secretion signal and C-terminal His_6_). We call this construct full-length Olfm1, although the C-terminal VIRSDEL segment is not included. 6 days after transient expression in *N*-acetylglucosaminyltransferase I-deficient Epstein-Barr virus nuclear antigen I-expressing HEK293 cells (U-Protein Express), medium was harvested and 5-fold concentrated using a 10-kDa molecular mass cutoff membrane. Protein was purified by nickel-nitrilotriacetic acid affinity chromatography followed by gel filtration on a Superdex200 column (GE Healthcare) equilibrated in gel filtration (GF) buffer (150 mm NaCl, 20 mm HEPES, pH 7.5). Protein was concentrated to 6 mg/ml using a 30-kDa molecular mass cutoff concentrator before plunge-freezing in liquid nitrogen.

##### SDS-PAGE and Western Blot

2 μl of purified protein was diluted with 8 μl of H_2_O and denatured by boiling with 5 μl of SDS loading dye with or without 6% (v/v) β-mercaptoethanol for reducing and non-reducing SDS-PAGE, respectively. For Western blot analysis, 10 μl of expression medium supernatant was denatured by boiling with 5 μl of (non-)reducing loading dye. Samples were run on standard Laemmli 12.5% (w/v) polyacrylamide Tris-glycine gels. Gels were either stained with Coomassie Blue or for Western blot blotted on a polyvinylidene difluoride (PVDF) membrane (Bio-Rad). Proteins were detected with a mixture of Qiagen mouse anti-penta-His, Sigma mouse anti-polyhistidine, and Dako rabbit anti-mouse HRP-conjugated antibodies.

##### Crystallization and Data Collection

Because initial crystallization attempts were fruitless, limited proteolysis by α-chymotrypsin was used for crystallization. α-Chymotrypsin (stock of 1 mg/ml in 1 mm HCl and 2 mm CaCl_2_) was added at 1:100 (v/v) to 6 mg/ml Olfm1 in 150 mm NaCl, 20 mm HEPES, pH 7.5. After 30-min incubation at room temperature, crystallization screens were set up using the sitting drop method by mixing 150 nl of protein-protease mixture (6 mg/ml) with 150 nl of reservoir solution. Crystals were grown at 20 °C in a condition containing 1 m LiCl, 20% PEG 6000 (w/v), and 100 mm Tris, pH 8.5.

Crystals from the original condition were cryoprotected with reservoir solution supplemented with 25% (v/v) glycerol and plunge-cooled in liquid nitrogen. A data set was collected at 100 K at the European Synchrotron Radiation Facility beamline ID23-1. Crystals diffracted to 2.4 Å. Data were integrated with iMOSFLM (18) and scaled and merged with Pointless/Aimless/Ctruncate (CCP4 suite) (19, 20).

##### Structure Solution and Refinement

The structure was solved by molecular replacement with the gliomedin β-propeller crystal structure (Protein Data Bank code 4D77 (14)) using Phaser (21). Subsequent rounds of manual model building and refinement were performed with Coot (22) and REFMAC5 (23). Final refinement was performed with PHENIX (24), and validation was performed with MolProbity (25). A large portion, 209 residues of a total of 463 residues for the mature full-length protein, is missing in the electron density most likely due to α-chymotrypsin treatment of Olfm1. The final model consists of residues Val^211^–Phe^477^ and Arg^210^–Ala^480^ (the last two alanines Ala^479^ and Ala^480^ are from the NotI restriction site) for chains A and B, respectively, excluding loops Ala^339^–His^352^ in both chains.

##### Size Exclusion Chromatography with Multiangle Light Scattering (SEC-MALS)

SEC-MALS was performed at room temperature using an analytical Superdex200 5/150 column (GE Healthcare) equilibrated with GF buffer. SEC was performed with online static light scattering (miniDAWN TREOS, Wyatt Technology) and a differential refractive index (Shimadzu RID-10A) on an ÄKTAmicro system equipped with a triple wavelength UV detector (GE Healthcare). Data were analyzed using the ASTRA software suite (Wyatt Technology). The differential refractive index signal was combined with the light scattering to determine the molecular mass using standard protocols. A d*n*/d*c* of 0.178 was calculated for Olfm1 based on eight predicted *N*-linked glycans. Conalbumin was injected at 10 mg/ml as a control and calibration standard (for conalbumin, a d*n*/d*c* of 0.185 was used).

##### Small Angle X-ray Scattering (SAXS)

SAXS was performed at the European Synchrotron Radiation Facility BM29 BioSAXS beamline equipped with a 2D Pilatus 1M detector (DECTRIS, Switzerland) operated at an energy of 12.5 keV. Full-length Olfm1 was diluted with and dialyzed against GF buffer using a 10-kDa molecular mass cutoff membrane. The concentration of Olfm1 was determined by UV-visible spectroscopy on a nanodrop ND-1000 spectrophotometer to be 3.55 mg/ml. SAXS data were collected at 20 °C. 18 successive 0.056-s frames were collected. The data were radially averaged and normalized to the intensity of the transmitted beam, exposure time, and sample concentration, and the scattering of the solvent blank (GF buffer) was subtracted. The curve was scaled using a BSA reference so that the *I*_0_ represents the Olfm1 molecular mass. Radiation damage was monitored by comparing curves collected on the same sample; no evidence for radiation damage was observed. Data were analyzed by PRIMUS (26), GNOM (27), and DAMMIF (28) of the ATSAS suite (29).

##### Analytical Ultracentrifugation (AUC)

Full-length Olfm1 was dialyzed against GF buffer using a 10-kDa molecular mass cutoff membrane. Protein was diluted with GF buffer to a concentration of 1.71 mg/ml. AUC sedimentation velocity was performed in a Beckman Coulter Proteomelab XL-I analytical ultracentrifuge using a 3-mm centerpiece, quartz windows, and an An-60 Ti rotor (Beckman). Absorption measurements were made at 42,000 rpm and 20 °C every minute at 280-nm wavelength and with GF buffer as reference. v̄, buffer density, and viscosity were determined by SEDNTERP to be 0.71006 ml/g, 0.99823 g/ml, and 0.001002 pascal·s respectively. Measurements were analyzed by SEDFIT using continuous *c*(s) mode (30, 31).

##### Thermofluor Stability Assay (TSA)

Thermofluor stability assays were performed using full-length Olfm1 diluted with TSA buffer (20 mm NaCl, 50 mm HEPES, pH 7.5) to concentrations of 1.2 mg/ml. Diluted protein or GF buffer was mixed 1:1 with 125× diluted SYPRO Orange (Sigma-Aldrich) in TSA buffer. 12.5 μl of SYPRO-protein mixture was mixed with a 12.5-μl volume consisting of 1, 5, or 20 mm CaCl_2_ or EDTA in TSA buffer or just TSA buffer. Denaturing curves were recorded on a MyiQ real time PCR thermocycler (Bio-Rad). A temperature ramp of 288–369 K was performed at 3 K/min. All measurements were performed in triplicate; curves were blank-subtracted, baseline-corrected, normalized to maximum fluorescence, and averaged.

##### Negative Stain Electron Tomography

Full-length Olfm1 was diluted with Milli-Q water to a concentration of 65 μg/ml. Carbon-coated mesh copper grids (Electron Microscopy Sciences, CF200-Cu) were glow-discharged for 15 s before incubation with protein for 30 s. Excess protein was wicked away with filter paper before grids were briefly washed two times with 5 μl of Milli-Q water and then stained for 30 s with a freshly prepared filtered 2% uranyl formate solution.

Electron tomography (ET) was performed with a Tecnai F20 (FEI Co.) at 200 kV, and images were acquired with a Gatan Ultrascan 4000 camera (Gatan Inc.). Tomographic tilt series were collected at a nominal magnification of ×30,000 with a final pixel size of 4.57 Å/pixel after ×2 binning. Tomograms were processed using IMOD (32), and phase contrast transfer function correction was performed on the tilt series using IMOD prior to reconstruction by weighted back-projection.

Subtomogram particles were manually picked using e2spt_boxer.py from EMAN2 (33). Each particle was normalized and masked with a sharp spherical mask to remove background density not associated with the protein. Particles were then filtered to 20 Å with a low pass Gaussian filter before a tight mask was applied to the remaining density. An Olfm1 coiled coil-β-propeller dimer was modeled using the crystal structure of Olfm1^coil-Olf^ and the homodimeric parallel coiled coil from myosin-V (Protein Data Bank code 2DFS (34)) to the predicted length of the Olfm1 coiled coil (see Fig. 3), adding full *N*-linked glycans to all predicted positions. Models were fitted manually by treating each Olfm1 dimer as one rigid body using UCSF Chimera (35).

## Results

### 

#### 

##### Olfm1 Forms Disulfide-linked Homotetramers

Recombinantly expressed Olfm1 lacking the C-terminal endoplasmic reticulum retention signal was purified from HEK293 supernatant for structural studies. Size exclusion chromatography as part of the purification strategy suggested a defined oligomeric assembly larger than a trimer. Analysis by reducing and non-reducing SDS-PAGE showed that this oligomer was disulfide-linked as seen by others (17, 36) (Fig. 1*B*).

The oligomeric assembly was independently shown to be tetrameric by SEC-MALS, AUC sedimentation velocity, and SAXS (Fig. 1, *C–F*). In SEC-MALS, a mass of ∼260 kDa was determined from the light scattering signal (Fig. 1*C*). This correlates well with the theoretical mass of 256 kDa for a fully glycosylated tetrameric Olfm1 (without any glycans the mass of a tetramer would be 217 kDa). In AUC, the tetramer had a sedimentation coefficient of 7.67 S and a frictional ratio *f*/*f*_0_ of 1.98, indicating an extended shape and corresponding to a molecular mass of 242 kDa (Fig. 1*D*). Further confirmation of a concentration-independent tetramer came from SAXS (Fig. 1*E* and Table 1); the molecular mass estimated from the *I*_0_ value of the Guinier plot of 249 kDa indicated a tetramer (Fig. 1*F*). The presence of monomers, dimers, or higher oligomeric species was not observed in any of these experiments.

**TABLE 1:** **SAXS parameters** ESRF, European Synchrotron Radiation Facility; SASBDB, Small Angle Scattering Biological Data Bank.

*I_0_* (normalized and referenced)	249
*R_g_* from Guinier plot (nm)	8.5
*R_g_* from *P*(*r*) (nm)	8.8
*D*_max_ from *P*(*r*) (nm)	30
*V*_porod_ (nm^3^)	616
Beamline	ESRF BM29
Protein concentration (mg/ml)	3.55
SASBDB accession code	SASDAS7

To show that the sole presence of Olfm1 tetramers was not an artifact of purification, Western blotting with α-His antibody was performed directly on the expression medium. This confirmed the presence of a disulfide-linked tetramer but showed no bands for other disulfide-linked oligomers (Fig. 1*B*).

##### Crystal Structure of a Covalent Dimeric Olfm1^coil-Olf^

Crystallization attempts of the full-length protein yielded no crystals, so we used limited proteolysis by α-chymotrypsin as a crystallization aid to remove unstructured regions. This yielded diffraction quality crystals and a data set to 2.4-Å resolution (see Table 2). Crystal structures of the olfactomedin domain of gliomedin (14) allowed us to do molecular replacement.

**TABLE 2:** **Crystallographic data collection and refinement** The highest resolution shell is shown in parentheses. ESRF, European Synchrotron Radiation Facility; CC, correlation coefficient.

	Olfm1^coil-Olf^
**Data collection**	
Beamline	ESRF ID23-1
Wavelength (Å)	0.97242
Unit cell parameters (Å; °)	*a* = 160.2, *b* = 43.94, *c* = 104.1; β = 114.2
Space group	C2
Resolution (Å)	50.3-2.4 (2.5-2.4)
No. of reflections	26,050 (2,925)
*R*_merge_ (%)	9.6 (93.6)
*I*/σ*I*	7.7 (1.2)
Completeness (%)	98.9 (99.4)
Redundancy	3.4 (3.5)
CC_1/2_ (%)	99.6 (48.9)
Wilson *B* factor (Å^2^)	49.9

**Refinement**	
*R*_work_/*R*_free_ (%)	24.1/25.8
Root mean square deviation	
Bond lengths (Å)	0.005
Bond angles (°)	0.884
No. of atoms/asymmetric unit	4,304
MolProbity score (percentile)	96th
Ramachandran most favored (%)	97
Ramachandran outliers (%)	0
Average *B* factor (Å^2^) (all atoms)	69.0
Protein chains/asymmetric unit	2
Protein Data Bank code	5AMO

**FIGURE 2.**
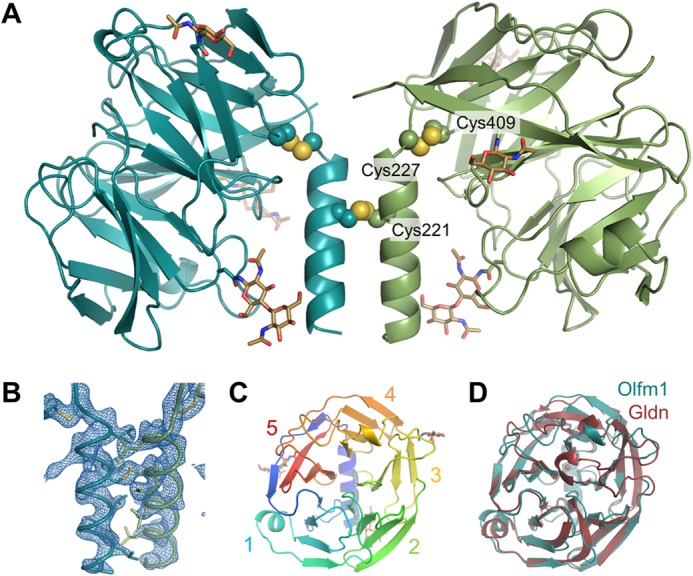
*A*, structure of the Olfm1^coil-Olf^ dimer. The inter- and intraprotein disulfides (shown as *spheres*) link the two monomers together and lock the β-propellers to the coiled coil, respectively. *B*, close-up view of the coiled coil and disulfides of the Olfm1^coil-Olf^ dimer showing the hydrophobic side chains in the coiled coil as *sticks*. The 2*F_o_* − *F_c_* electron density map was plotted at 1.2σ. *C*, view down the top face of a single β-propeller shows five blades, which are numbered accordingly. *D*, the structure of the olfactomedin domain of gliomedin (*Gldn*; *red*) is very similar to the olfactomedin domain of Olfm1 (*teal*; root mean square deviation of 1.3 Å over 226 aligned Cα atoms).

**FIGURE 3.**
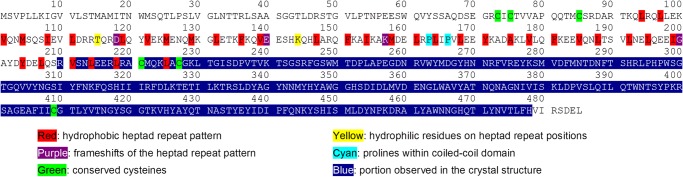
**Analysis of the hydrophobic heptad repeat pattern in the coiled-coil domain of Olfm1.** The full amino acid sequence of mouse Olfm1 is shown. Hydrophobic heptad repeat residues have been indicated based on the register of coiled-coil residues 210–225, which were observed in the crystal structure. Also indicated are possible disturbances from an ideal coiled coil such as frameshifts, hydrophilic residues on heptad repeat positions, and prolines in the coiled coil.

The structure revealed a disulfide-linked dimer of β-propellers in the asymmetric unit related by a pseudo-2-fold rotation of 178° (Fig. 2*A*). As expected, each β-propeller corresponds to a C-terminal olfactomedin domain. In addition to the β-propellers, a segment of the coiled-coil domain (residues Val^211^–Leu^225^) was visible in the electron density (Fig. 2*B*). Cys^221^ in this coiled-coil segment forms an interprotein disulfide, covalently linking the two β-propellers. The coiled coil is predicted to be longer (Fig. 3), but we did not observe additional residues N-terminal of Val^211^. Most likely, the segment of coiled coil absent from the crystals was hydrolyzed by α-chymotrypsin prior to crystallization. The Val^211^–Leu^225^ segment appears to be inaccessible for proteolysis due to steric hindrance by the two β-propellers. The dimer contacts are exclusively formed by the coiled coils; the two β-propellers are not contacting each other directly. Cys^227^, the first residue of the β-propeller, forms an intraprotein disulfide with Cys^409^ as predicted (15); this disulfide bond appears to close the β-propeller fold and lock it in a fixed orientation with respect to the coiled coil (Fig. 2, *A* and *B*). The β-propeller is tilted with respect to the coiled coil with an angle of 30° between the β-propeller plane and the coiled-coil axis. Hence, the two β-propellers in the covalent dimer have a 60° angle between each other, resulting in an exposed outward orientation of the β-propeller top faces.

##### The Olfm1 β-Propeller

The olfactomedin domain of Olfm1 forms a five-bladed β-propeller similar in structure to the recently solved olfactomedin domains of gliomedin, myocilin, and latrophilin3 (14–16). Each of the five blades in Olfm1 consists of four β-strands. The N-terminal segment of the β-propeller (Lys^229^–Thr^241^) forms a β-strand that completes the fifth β-propeller and closes the β-propeller fold (in addition to the intramolecular disulfide bond Cys^227^-Cys^409^; Fig. 2, *A*, *B*, and *C*). The two β-propellers in the dimer are very similar in structure (root mean square deviation of 0.4 and 0.9 Å over all Cα atoms and all atoms, respectively) with largest differences in the N and C termini.

We compared the olfactomedin domain of Olfm1 with those of gliomedin (14) and latrophilin3 (16) (Protein Data Bank codes 4D77 and 5AFB, respectively). The three olfactomedin domain structures are very similar to each other with a root mean square deviation of 1.3 Å for gliomedin and 1.1 Å for latrophilin3 over 226 aligned Cα atoms (Figs. 2*D* and 5*A*). These scores are also reflected in the respective sequence identities of 32 and 42% for the olfactomedin domain. Largest differences are apparent in three loops at the top face of the β-propeller (within and between blades 3, 4, and 5) and in one loop in blade 1 at the β-propeller bottom face that interfaces with the coiled coil in Olfm1.

**FIGURE 4.**
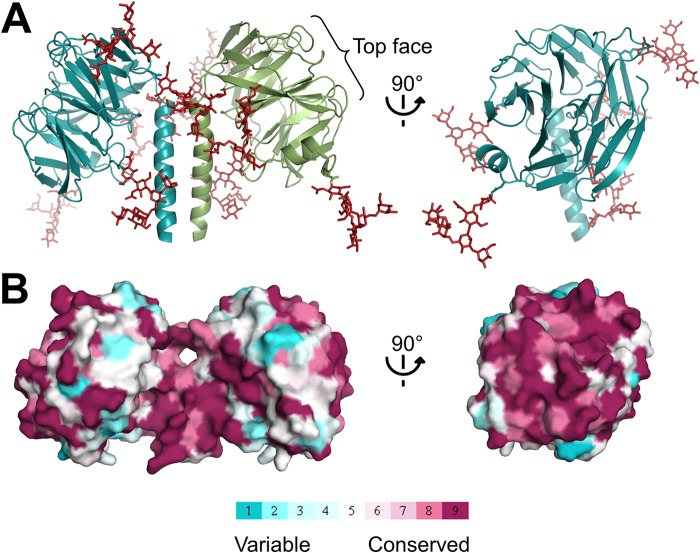
*A*, Olfm1^coil-Olf^ dimer with modeled full glycans (*red*) and longer coiled coils shows that all glycans are localized to the side and bottom face of the β-propeller, whereas the top face is accessible. *B*, conservation plot indicates that the top face of the β-propeller has a higher degree of sequence conservation than the sides, suggesting that this interface might be important for ligand binding. Conservation scores were calculated with an alignment of 35 vertebrate Olfm1 orthologs (supplemental Fig. 1) using ConSurf (42).

Most likely, all of the five predicted *N*-linked glycosylation sites in the β-propeller (Asn^288^, Asn^307^, Asn^394^, Asn^431^, and Asn^473^) are glycosylated as we observed at least some density for the five glycans in one of the two β-propellers. Remarkably, all glycosylation sites are located at either the side or the bottom of the β-propellers, whereas the top faces are free from glycans and completely exposed (Fig. 4*A*). Furthermore, the top face of the β-propellers is evolutionarily more conserved than the sides or the bottom (Fig. 4*B*). These observations together with an exposed outward orientation suggest a role for the β-propeller top face in ligand binding.

##### Calcium Stabilizes Olfm1

**FIGURE 5.**
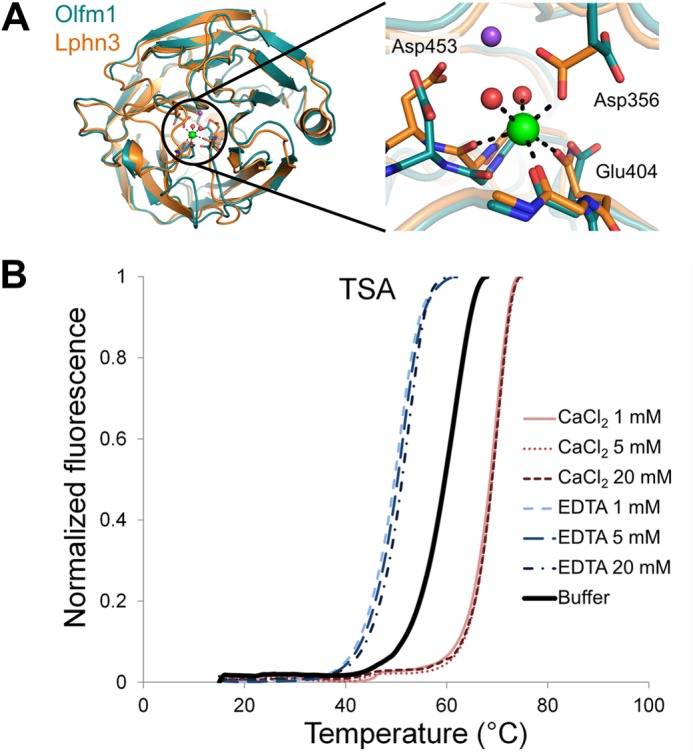
*A*, comparison of the calcium-binding olfactomedin domain of latrophilin3 (*Lphn3*; *orange*) and Olfm1 (*teal*). It is likely that Olfm1 can also accommodate a calcium ion at the center of the β-propeller. The latrophilin3-coordinated calcium ion (*green*) and neighboring sodium ion (*purple*) are shown. *B*, TSA shows a pronounced stabilizing effect of Olfm1 by calcium, shifting the melting temperature 8 °C higher, whereas EDTA destabilizes Olfm1 (a shift to 10.5 °C lower). *Buffer* indicates TSA buffer without calcium or EDTA added.

A cation binding site is present in the center of the olfactomedin domain β-propeller of gliomedin (14), myocilin (15), and latrophilin3 (16). In myocilin and latrophilin3, a calcium ion and a sodium ion are located next to each other in this site, whereas in gliomedin, one sodium ion is present. The Olfm1 cation binding site resembles those of myocilin and latrophilin3; two of the three cation-coordinating side chains are identical in the three proteins (Asp^356^ and Asp^453^; Olfm1 residue numbering), and one, Glu^404^, is replaced by Asn in myocilin and latrophilin3 (Fig. 5*A*). We observed a positive 5σ peak at this site in the *F_o_* − *F_c_* difference electron density map after initial refinement but cannot confidently place a calcium ion here even at lowered occupancies. Thermal denaturation assays showed that excess calcium stabilized the protein at concentrations as low as 1 mm, whereas EDTA destabilized it (Fig. 5*B*). Based on cation binding site similarity to myocilin and latrophilin3 and our observation that calcium stabilizes Olfm1, we hypothesize that Olfm1 also has a calcium binding site located at this site in the center of the β-propeller.

##### The Olfm1 Tetramer Is Arranged as a V-shaped Dimer of Dimers

**FIGURE 6.**
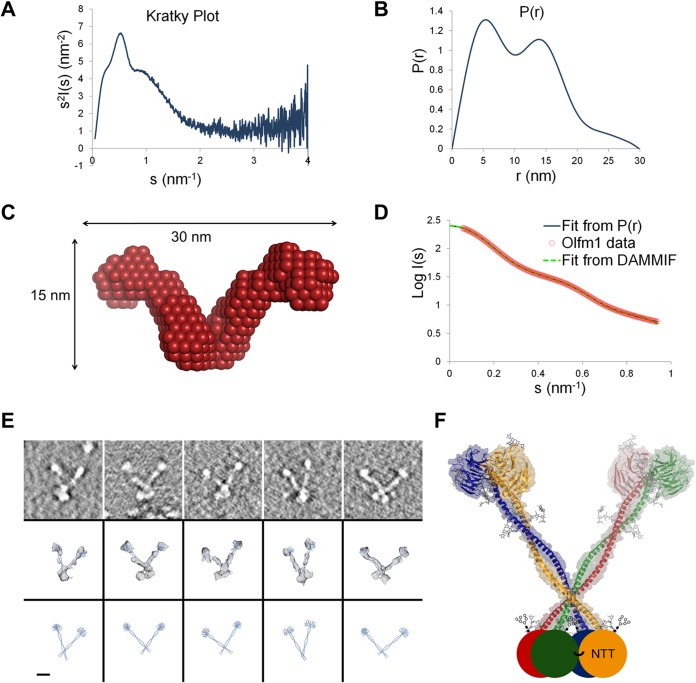
**The full-length Olfm1 tetramer has a V-shaped architecture.** *A*, the Kratky plot shows no signs of large unstructured regions (37). *B*, the SAXS pair distance distribution function *P*(*r*) indicates a dumbbell-like shape. *C*, *ab initio* modeling by DAMMIF with imposed 2-fold rotational symmetry reveals a V-shaped architecture. A single (non-averaged) DAMMIF model is shown that is representative for the average structure generated from 50 models without applying 2-fold symmetry. *D*, fit from the *P*(*r*) and the DAMMIF *ab initio* modeling (χ^2^ = 4.313). *E*, ET shows V shapes with varying angles between the legs. The dimensions of the coiled coil and the double β-propeller from the crystal structure fit those of the ET densities. *Top panels*, central slice through the tomograms showing the V shapes. *Middle panels*, fit of two double β-propellers with modeled coiled-coil domains in the ET density. *Bottom panels*, same as *middle panels* but without the ET densities. *Scale bar*, 10 nm. *F*, model of the full-length Olfm1 tetramer. Each monomer is represented by a different color. In the NTT domains, one of the interprotein disulfides necessary for tetramerization is indicated.

Analysis of the full-length Olfm1 tetramer in solution by SAXS and by negative stain ET indicated that Olfm1 has a V-shaped architecture. The SAXS data show that Olfm1 has a rather rigid structure as the Kratky plot has low values at higher scattering angles (37) (Fig. 6*A*). The pair distance distribution function *P*(*r*) remarkably shows two maxima at 5 and 14 nm (Fig. 6, *B* and *D*), indicative of a dumbbell-like shape. *Ab initio* modeling by DAMMIF (28) with imposed 2-fold rotational symmetry suggested a V-shaped arrangement (Fig. 6, *C* and *D*). Likewise, ET analysis revealed a V-shaped architecture with varying angles between the legs. A bilobal shape was observed at the base of the “V” in the tomograms that is likely formed by the N-terminal tetramerization region (Fig. 6*E*). This suggests that this region forms a folded domain, which we refer to as the N-terminal tetramerization (NTT) domain.

In the DAMMIF bead model, the two tips of the V are separated by 21 nm, whereas in the ET maps, this distance varies from 13 to 28 nm. The *D*_max_ from SAXS indicates a maximum distance of 30 nm in the Olfm1 tetramer, correlating with the maximum distance from the ET data of ∼32 nm. Thus, we observed a V-shaped architecture for the full-length Olfm1 tetramer both in SAXS and ET experiments with similar dimensions for the tetramer between the two techniques (Fig. 6).

The V shape of Olfm1 agrees well with our crystal structure of the dimeric Olfm1^coil-Olf^ and with our data on tetramerization of Olfm1. The two tips of the V legs each appear as a bulky feature in both the SAXS and ET data and are readily identifiable as one β-propeller dimer each. The V legs are both formed by a coiled-coil dimer, whereas the base of the V is formed by four copies of the NTT domain (Fig. 6*F*). This tetramer of NTT domains is arranged in a bilobal shape and harbors cysteines Cys^73^ and Cys^75^ shown previously to be essential for oligomerization (17). It is noteworthy that we constructed this model with a dimeric β-propeller at the tips of the V shape before we solved the crystal structure. The dimeric Olfm1^coil-Olf^ structure essentially validated this model.

## Discussion

### 

#### 

##### Tetramerization

Although others have shown that Olfm1 forms covalent oligomers *in vivo*, the stoichiometry of the assembly has not hitherto been reported. We show that Olfm1 consistently forms stable covalent tetramers and is not further covalently oligomerized as was suggested previously by others (9, 36). Interestingly, myocilin also has a coiled coil N-terminal of the olfactomedin domain, and others have shown that it forms covalent dimers and tetramers (38). Similarly, gliomedin has a trimerizing collagen-like helix N-terminal of its olfactomedin domain (39). It has been shown that Olfm1 co-purifies with Olfm2, which also co-purifies with Olfm3 (12). It is not clear whether these strongly related paralogs form mixed heterotetramers with a similar arrangement or whether they interact non-covalently. Nonetheless, we have shown that Olfm1 can form stable homotetramers and likely also does so *in vivo*.

##### V-shaped Conformation

The combination of our ET data, the *P*(*r*) and *ab initio* modeling by SAXS, and the crystal structure of the Olfm1^coil-Olf^ dimers agrees with a V-shaped dimer-of-dimers architecture for the full-length Olfm1 tetramer. The ET data suggest some flexibility of the two legs of the V, which may be caused by two proline residues found in a P*XX*P motif at Pro^163^ and Pro^166^ within the coiled coil. Although these do not interrupt the heptad repeat pattern of the coiled coil (Fig. 3), it is known that prolines disrupt hydrogen bonding within α-helices, allowing a kink in the rodlike coiled coil and affecting the overall V shape of the tetramer. In addition, there may be flexibility in the connection between the NTT domains and the coiled coils that affects the angle between the V legs. Cys^73^ and Cys^75^, which are located in the NTT domain, have been shown previously to be essential for oligomerization (17). The ET data show a bilobal shape at the base of the V, suggesting that the NTT domains are also arranged as a dimer of dimers. However, the detailed structure of the NTT domain and a portion of the coiled coil remain elusive.

##### Calcium Binding Site

Olfm1 has not been described previously as a calcium-binding protein. It has a site similar to the myocilin and latrophilin3 calcium binding site consisting of two aspartates (Asp^356^ and Asp^453^) and a glutamate (Glu^404^). These residues are the same in Olfm1 paralogs Olfm2 and Olfm3, whereas Glu^404^ and Asp^453^ are both asparagine in Olfm4. TSA showed that excess calcium indeed stabilized Olfm1, whereas EDTA was destabilizing. Most likely, Olfm1 contains a calcium binding site as do Olfm2 and Olfm3. Whether the calcium is only structurally stabilizing the protein or serves a regulatory purpose remains to be determined.

##### Other Olfactomedins Are Possibly V-shaped

**FIGURE 7.**
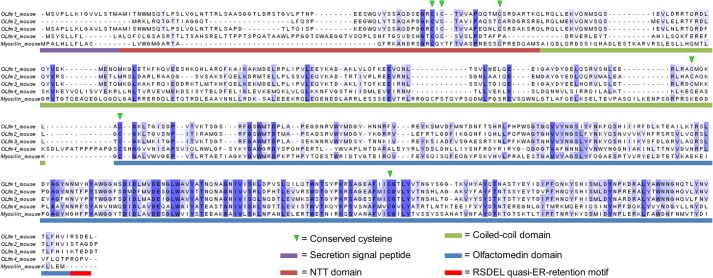
**Sequence alignment of Olfm1 with the closely related (mouse) paralogs Olfm2, Olfm3, Olfm4, and myocilin.** Domain boundaries are indicated with the same color scheme as in Fig. 1*A*. Conserved cysteines are indicated by *arrowheads*. Residues are colored according to the percentage of sequence identity (*blue* means conserved; *white* means variable). *ER*, endoplasmic reticulum.

The V-shaped tetrameric conformation we observed for full-length Olfm1 is possibly adopted by several other olfactomedin family members. Sequence analysis indicates that the features that define this architecture, two or more cysteines near the N terminus, a central coiled-coil domain, and a C-terminal β-propeller, are also present in Olfm2, Olfm3, Olfm4, and myocilin(Fig. 7). In addition, the Olfm1 paralogs (Olfm2, -3, and -4) also have a cysteine equivalent to Cys^221^ in Olfm1 that covalently links the C-terminal end of the coiled-coil domain and two β-propellers. Myocilin does not have a cysteine in this region, and possibly the β-propellers in myocilin have more flexibility with respect to the coiled coil. Taken together, the data suggest that the V-shaped conformation with two β-propeller dimers attached to each other via a coiled-coil domain is more common within the olfactomedin superfamily.

##### Functional Importance of Architecture

The striking architecture of Olfm1, encompassing two exposed and substantially separated β-propeller dimers with conserved top faces, is likely important for function. Olfm1 interacts with a diverse set of proteins, many of which are cell surface-attached receptors. Possibly, Olfm1-receptor interactions are mediated by the β-propellers (*i.e.* the olfactomedin domains), although for Nogo receptor 1, it has been shown that interaction does not rely on the olfactomedin domain (6). In this mode, Olfm1 can engage multiple receptor molecules simultaneously and may bring receptors together or induce receptor clustering to regulate signaling.

The oligomeric state of many olfactomedin family members has been shown to be important for function (10). Besides Olfm1–4 and myocilin, which are likely to form tetramers with separated β-propellers, gliomedin also has substantially separated β-propellers in a trimerized form (14). Latrophilin3, however, is not known to oligomerize and lacks a coiled-coil or collagen domain. The binding and signaling mode among Olfm1–4, myocilin, and gliomedin is possibly related and as we hypothesize for Olfm1 may involve oligomerization or clustering of receptors.

##### Possible Role in Cation Channel Formation, Stabilization, or Regulation

Olfm1 has been reported to be a binding partner of cation channels such as the AMPA receptor complex and the Cav2.1 voltage-gated calcium channel. Moreover, genetic deletion of a part of the coiled-coil region leads to elevated calcium concentrations in the cytosol as well as developmental and behavioral defects (8). Because both the AMPA receptor and Olfm1 form homotetramers with a 2 × 2 arrangement, it is tempting to hypothesize that Olfm1 plays a role in AMPA receptor complex stabilization or regulation. Alternatively, an Olfm1 tetramer may bind two or even four AMPA receptor complexes and play a role in the supramolecular organization of these complexes by providing a scaffold for formation of larger clusters of AMPA receptors.

Olfm2 has been shown to have a similar role in AMPA receptor binding or regulation (11). Gliomedin is also known to be involved in sodium channel clustering and maintenance (40, 41). Remarkably, whereas Olfm1 and Olfm2 bind calcium channels and likely a calcium ion within the β-propeller, gliomedin binds sodium channels and sodium in its β-propeller. It is noteworthy that all of them seem to share a cation binding site and an oligomerization domain N-terminal of their olfactomedin domains. Whether other olfactomedin domain-containing proteins such as Olfm3 or myocilin could have a similar role in ion channel stabilization or regulation needs further investigation.

In conclusion, we have shown that Olfm1 forms disulfide-linked homotetramers with a V-shaped architecture and provided high resolution data for the C-terminal Olfm1^coil-Olf^. This sheds light on the structure and quaternary organization of full-length Olfm1 as well as family members and provides new insights into function.

## Supplementary Material

Supplemental Data

## Footnotes

2 The abbreviations used are:

Olfm: olfactomedin
GF: gel filtration
SEC-MALS: size exclusion chromatography with multiangle light scattering
SAXS: small angle x-ray scattering
AUC: analytical ultracentrifugation
TSA: Thermofluor stability assay
ET: electron tomography
NTT: N-terminal tetramerization.
