# Supplementary material for: Olfactomedin-1 Has a V-shaped Disulfide-linked Tetrameric Structure
Source: J Biol Chem. 2015 Apr 21;290(24):15092–101. doi: 10.1074/jbc.M115.653485 (PMC4463452; doi:10.1074/jbc.M115.653485)
Supplement: Supplemental Data [file supp_290_24_15092__index.html]

Olfactomedin-1 has a V-shaped disulfide-linked tetrameric structure. — Olfactomedin-1 Has a V-shaped Disulfide-linked Tetrameric Structure — Structural Characterization of the Olfactomedin-1 Tetramer — Supplemental Data 

# Olfactomedin-1 Has a V-shaped Disulfide-linked Tetrameric Structure

## Supplemental Data

**Files in this Data Supplement:**

- Supplemental Figure 1 (.pdf, 1.1 MB) - Multiple sequence alignment of 35 vertebrate Olfactomedin-1 orthologs
