# Supplementary material for: Olfactomedin-1 Has a V-shaped Disulfide-linked Tetrameric Structure
Source: J Biol Chem. 2015 Apr 21;290(24):15092–101. doi: 10.1074/jbc.M115.653485 (PMC4463452; doi:10.1074/jbc.M115.653485)
Supplement: Supplemental Data [file supp_M115.653485_jbc.M115.653485-1.pdf]

## Supplemental Figure 1

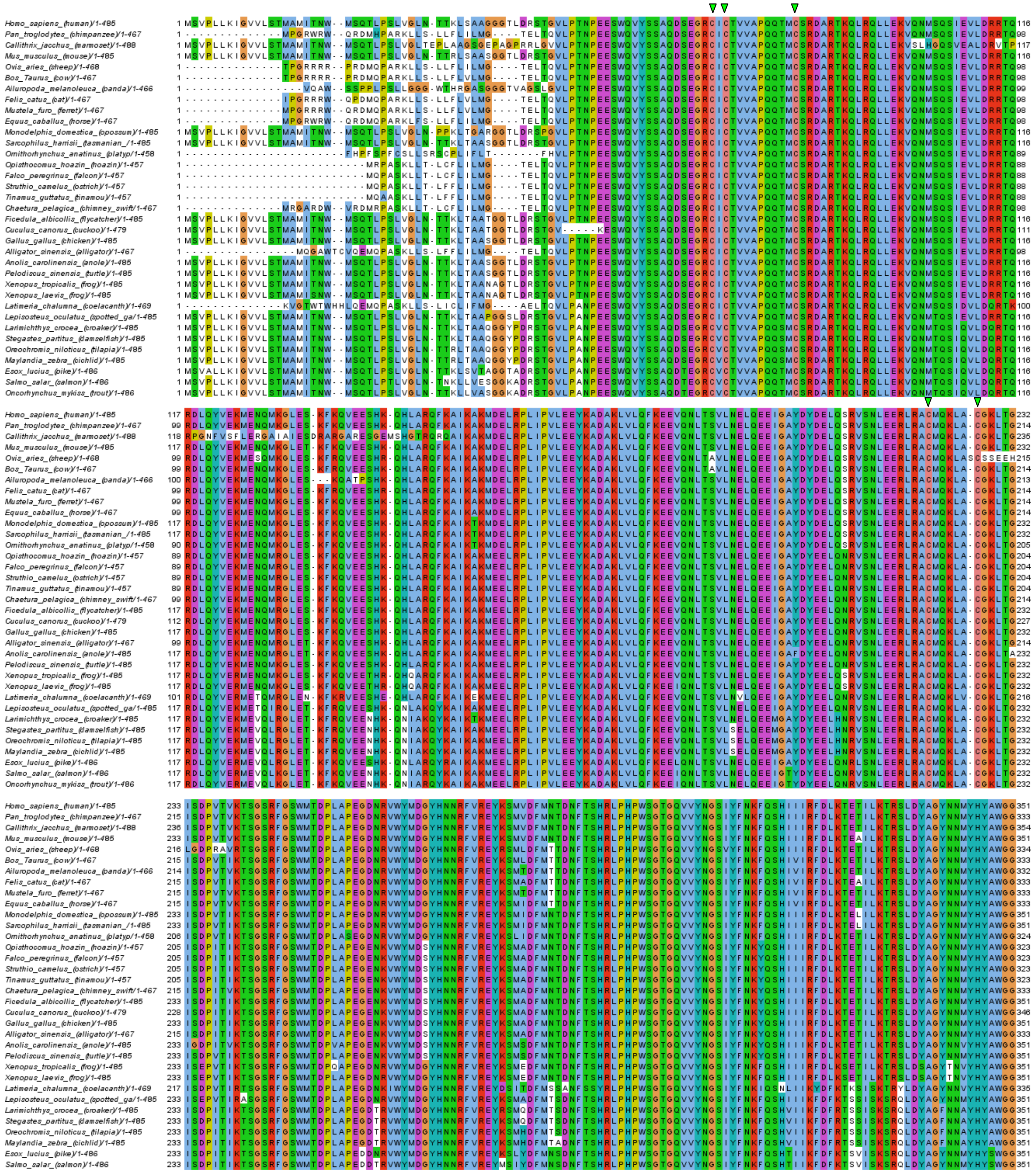

▼ = Conserved cysteine

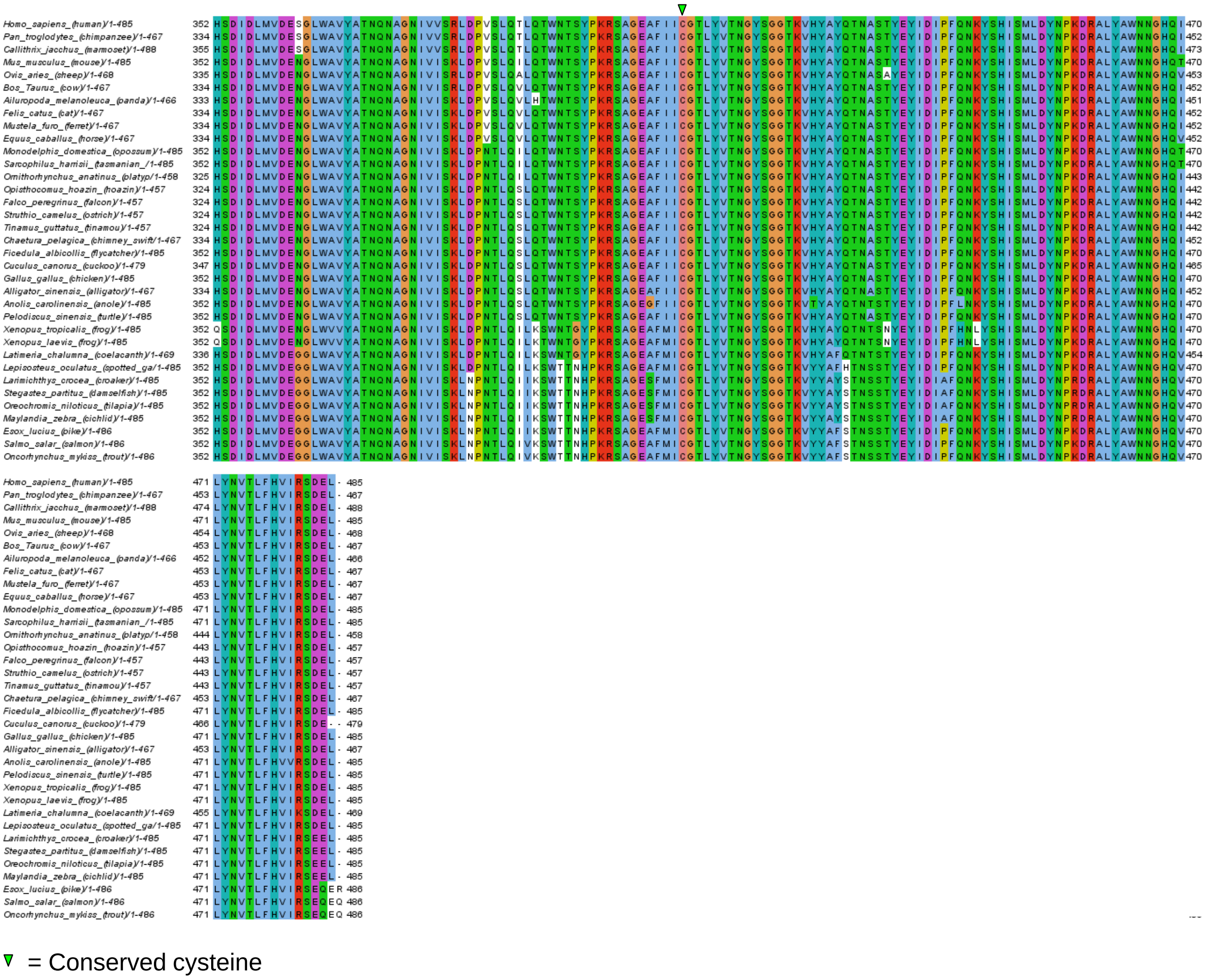


**Supplemental figure 1**

Multiple sequence alignment of 35 vertebrate Olfactomedin-1 orthologs. Conserved cysteines are indicated by arrowheads. This alignment was used for the Consurf analysis (Fig. 4B).
